# Supplementary material for: β-Carotene accelerates the resolution of atherosclerosis in mice
Source: eLife. 2024 Feb 6;12:RP87430. doi: 10.7554/eLife.87430 (PMC10945528; doi:10.7554/eLife.87430)
Supplement: Supplementary file 1. [file elife-87430-supp1.docx]

**Supplementary File 1**

**Supplementary Table 1.** Composition of the experimental diets utilized in the study.

| **Ingredient** | **WD-**  **VAD**  **(g/kg diet)** | **WD-**  **β-carotene**  **(g/kg diet)** | **Standard-**  **VAD**  **(g/kg diet)** | **Standard-**  **β-carotene**  **(g/kg diet)** |
| --- | --- | --- | --- | --- |
| **Casein** | 200 | 200 | 200 | 200 |
| **L-Cysteine** | 3 | 3 | 3 | 3 |
| **Corn starch** | 72.8 | 72.8 | 319 | 319 |
| **Maltodextrin** | 100 | 100 | 100 | 100 |
| **Sucrose** | 212 | 212 | 212 | 212 |
| **Cellulose** | 50 | 50 | 50 | 50 |
| **Soybean oil** | 25 | 25 | 70 | 70 |
| **Lard** | 160 | 160 | 0 | 0 |
| **t-Butylhydroquinone** | 0 | 0 | 0 | 0 |
| **Choline bitartrate** | 2 | 2 | 2 | 2 |
| **Dicalcium phosphate** | 13 | 13 | 13 | 13 |
| **Calcium carbonate** | 5.5 | 5.5 | 5.5 | 5.5 |
| **Potassium citrate monohydrate** | 16.5 | 16.5 | 16.5 | 16.5 |
| **Cholesterol** | 3.08 | 3.08 | 0 | 0 |
| **Mineral mix** | 10 | 10 | 35 | 35 |
| **Vitamin mix, no added vitamin A** | 10 | 10 | 10 | 10 |
| **Placebo beadlets** | 0.5 | 0 | 0.5 | 0 |
| **β-carotene beadlets, 10% β-carotene** | 0 | 0.5 | 0 | 0.5 |

# ^1^ Footnotes. IU: International unit. WD, Western diet; VAD; Vitamin A deficient; IU, international units.
